# A Laser-Engraving Technique for Portable Micropneumatic Oscillators

**DOI:** 10.3390/mi9090426

**Published:** 2018-08-24

**Authors:** Vidhya Balaji, Kurt Castro, Albert Folch

**Affiliations:** 1 Department of Electrical Engineering, University of Washington, Seattle, WA 98195, USA; 2 Department of Bioengineering, University of Washington, Seattle, WA 98195, USA; castrokb@uw.edu (K.C.); afolch@uw.edu (A.F.)

**Keywords:** microfluidic automation, laser engraving, manufacturability, pneumatic oscillator, APTES surface activation

## Abstract

Microfluidic automation technology is at a stage where the complexity and cost of external hardware control often impose severe limitations on the size and functionality of microfluidic systems. Developments in autonomous microfluidics are intended to eliminate off-chip controls to enable scalable systems. Timing is a fundamental component of the digital logic required to manipulate fluidic flow. The authors present a self-driven pneumatic ring oscillator manufactured by assembling an elastomeric sheet of polydimethylsiloxane (PDMS) between two laser-engraved polymethylmethacrylate (PMMA) layers via surface activation through treatment with 3-aminopropyltriethoxysilane (APTES). The frequency of the fabricated oscillators is in the range of 3–7.5 Hz with a maximum of 14 min constant frequency syringe-powered operation. The control of a fluidic channel with the oscillator stages is demonstrated. The fabrication process represents an improvement in manufacturability compared to previous molding or etching approaches, and the resulting devices are inexpensive and portable, making the technology potentially applicable for wider use.

## 1. Introduction

Microfluidic automation can feature large arrays of valves and pumps performing multiple analyses for diagnostic assays and biological applications [1]. The development of complete systems that integrate sample preparation, fluidic manipulation, and detection mechanisms [2] is associated with increasingly complex interconnections and microvalve automation. Microfluidic very large-scale integration (mVLSI) [3] has enabled parallel fluidic manipulations using thousands of microvalves, thereby alleviating some of the burden. However, the associated control hardware requires many external connections, thereby making the management of its operation prone to errors (leaks, etc.) and cumbersome to operators unfamiliar with the technology. Designs that minimize the number of control signals [4] have provided an impetus to parallelization, but they have not eliminated entirely the challenge of external connection complexity.

In order to drastically reduce off-chip connections, researchers have explored the integration of microfluidic logic control elements on chip of varying complexity [5,6,7,8] with the aim of facilitating the development of simple-to-use, portable devices. These circuits have been fabricated with time and labor-intensive techniques such as soft lithography [9] and glass etching, limiting the manufacturability and mass production capability. The expansion of microfluidic circuits into the realm of point-of-care testing (POCT) hinges on the simplification of the manufacturing process and the automation of the steps involved [10]. To this end, computer numerical control (CNC) milling [11] and laser engraving [12] have been explored as techniques for microfluidics fabrication. Micro-milled three-dimensional microfluidic flow cells have demonstrated the potential of milling for medical diagnostics [13].

Laser-engraving has emerged specifically as a very promising technology due to factors such as fastest turnaround time, comparatively low operational costs and ease of design processing. A wide range of polymers can be processed by a laser cutter, including thermoplastics such as polymethylmethacrylate (PMMA). Thermoplastics are extensively used for microfluidics due to their high manufacturability and properties amenable to microfluidic analysis [14,15]. Valves and pumps have been demonstrated by sandwiching polydimethylsiloxane (PDMS) between channel structures created on PMMA [16].

Implementing self-driven microfluidic devices requires an on-chip timing reference or clock generation circuit [17]. Pneumatic oscillators fabricated with glass and PDMS have been characterized by Duncan et al. [7]. Scalability of microfluidic logic circuits using prototyping techniques such as CNC milling has also been shown [18]. In this paper, the authors demonstrate an all-plastic (PMMA/PDMS) ring oscillator chip with features manufactured using laser engraving and driven with a syringe which acts as a simple and portable pneumatic power source. The device is assembled by permanently bonding the three layers using chemical surface activation in contrast to mechanical approaches to keeping the layers together, such as binder clips [19] which cannot form a direct bond between the PDMS and PMMA [20].

## 2. Materials and Methods 

The ring configuration, wherein an odd number of NOT gates or inverters are connected in a feedback loop, is one of the standard structures for an oscillator used in semiconductor electronics. A fluidic equivalent is based on the analogy between the normally off n-type metal oxide semiconductor field effect transistor (N-MOSFET) operating with a voltage differential and a normally closed valve operating between two pressures—in this case, psi of vacuum (VAC) and atmosphere (ATM).

**Figure 1:**
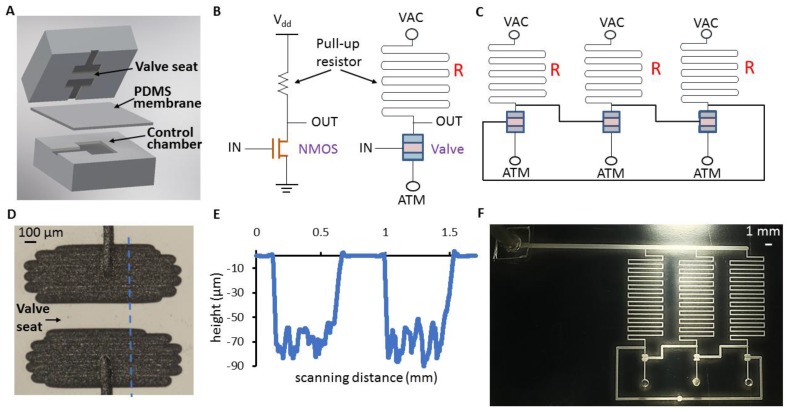
(**A**) Three-layer valve structure before assembly; (**B**) N-type metal oxide semiconductor (NMOS) inverter (**left**) along with micropneumatic equivalent (**right**). *V*_dd_, voltage supply in volts; VAC, vacuum supply in psi; ATM, atmospheric pressure; *R*, pull-up resistance; (**C)** Pneumatic ring oscillator with the connection between the output of the third inverter and the input of the first inverter being the feedback loop; (**D**) Laser-cut valve (top-view); (**E**) Height profile of the flow chambers and seat of the valve along the section denoted by the blue dotted line in (**D**); (**F**) Laser-cut ring oscillator with silicone tubes attached as vacuum supply inlet for testing the operation.

As shown in Figure 1, the normally closed valve consisting of a PDMS membrane sandwiched between a PMMA flow layer and a PMMA control layer is connected to the pressure supply (VAC) through a fluidic resistor to form a pneumatic inverter. A logic 0 at the input pulls the output to a 1 through the resistor, whereas an input of 1 opens the valve, driving the output to 0. Device layers of the three-stage pneumatic ring oscillator are fabricated by laser engraving (CO_2_ laser VersaLaser, Universal Laser Systems, Scottsdale, AZ, USA; 10.2 µm wavelength; 10–60 W power) in PMMA (Astari-Niagara, Jakarta, Indonesia; 0.125-in thick).

### 2.1. Parameters of Design and Assembly Process

Oscillation frequency is dependent on two main design parameters: (1) inverter stage resistance *R_s_*—the sum of valve, pull-up, and parasitic interconnect resistances, and (2) valve chamber volume *V_c_* (which directly impacts the pneumatic capacitance of the valve). The cross-section of the microfluidic channel representing the fluidic resistor is approximately rectangular with a designed dimension of 250 µm × 50 µm (width × height). The fluidic resistance of a rectangular channel is:

(1) $$\;R_{f}=\frac{12µL\;}{wh^{3}f}$$

where *h* is the height of the channel, *w* is the width, *L* is the length, µ is the viscosity, and *f* is the geometric form factor related to the rectangular shape. When *h* << *w*, *f*~1 does not influence the value of *R_f_*. Pressure supply (*P_s_*) is the operational parameter that dynamically influences the oscillator frequency. Theoretically, the time period is proportional to (*R_s_* × *V_c_*)/*P_s_*, analogous to the expression for the time constant of an RC circuit. The correlation with experimental values was confirmed upon testing the oscillators. The Poiseuille’s equation for compressible fluids provides an analytical relation between pressure, volumetric flow rate, and resistance through a pneumatic circuit with a channel and chamber:

(2) $$\;Q_{t}=\frac{P_{v}-P_{c}\;}{R_{ch}}\frac{P_{v}+P_{c}}{2P_{c}}$$

where *P_v_* is the vacuum supply, *P_c_* is the chamber pressure, and *R_ch_* is the fluidic resistance of the channel connecting the supply to the chamber. The fluidic resistance of the channel was calculated as per Equation (1). A 2D schematic of the oscillator (Autodesk Inventor Professional 2017, Autodesk, San Rafael, CA, USA) was drawn with pull-up resistors and valve sizes based on the targeted frequency. The line diagram (AutoCAD 2017, Autodesk, San Rafael, CA, USA) derived by identifying the center lines of symmetry of the channels and the valve sections was then used directly as input to the laser’s proprietary software. A calibration of the engraved line widths with different speed/power settings for a given schematic line width served as a reference to determine the appropriate feature dimensions for the oscillator schematic. For the range of channel widths used in the design, the raster mode of the laser was found to be suitable (laser speed: 20–40% of maximum; power: 18–20% of maximum). The consequent multiple scans of the laser-enhanced surface roughness indicated the profile of a valve flow chamber, as shown in Figure 1E. The feature depth considered for resistance calculations was the average depth of the profile. A Dektak profilometer (Bruker, Billerica, MA, USA) measured the dimensions of a single valve as 1.4 mm × 1.2 mm (Length × Width) with an average depth of 65 µm. 

Two or three rinses with isopropyl alcohol (IPA) followed by soaking in deionized water for approximately 10 min were required to remove leftover debris from the features on the PMMA surface after laser engraving. The assembly of the PMMA layers with PDMS film (Rogers HT-6240, 254 µm, Rogers, Woodstock, CT, USA) was accomplished in three steps: (1) Surface activation of PMMA with 3-aminopropyltriethoxysilane (99% APTES, Sigma-Aldrich, St. Louis, MO, USA). After trials of varying the concentration of APTES (1–5%), 5% solution heated to 85 °C provided the optimal outcome for bonding. Soaking the PMMA for 30 min in heated APTES solution was adequate; (2) O_2_ plasma treatment of the PDMS (Diener Zepto, Ebhausen, Germany, at 660 mTorr O_2_); and (3) Placing the PDMS on the PMMA in conformal contact for bonding and heating in an oven at 70 °C for 2 h. Via holes were created in the bonded PMMA-PDMS piece which was then sandwiched with the remaining PMMA layer after APTES and O_2_ plasma steps were carried out as described above. The assembly was then clamped between flat metal plates to ensure uniform pressure across the device area before placing it in the oven. Upon removal from the oven after 2 h, silicone tubes (Cole Parmer, Vernon Hills, IL, USA, 1.14 mm inner diameter) bonded to PDMS were attached to the vacuum inlet using a silicon-silicone adhesive tape (S1001-1DC11, Adhesive Applications Inc., Easthampton, MA, USA) to carry out testing.

The significance of using the APTES approach was the strong and permanent bond formed between the PMMA and PDMS. Comprehensive studies of the bonding of PMMA and PDMS substrates have shown that reliable and reproduceable results are possible with this technique [21]. The experiments carried out for this study confirmed this observation after the procedure was standardized for surface activation and assembly. Additionally, the bond strength of the fabricated devices remained unchanged within a time duration of a few months, demonstrating the structural stability required for use in portable and hand-held application scenarios.

### 2.2. Characterization Technique

**Figure 2:**
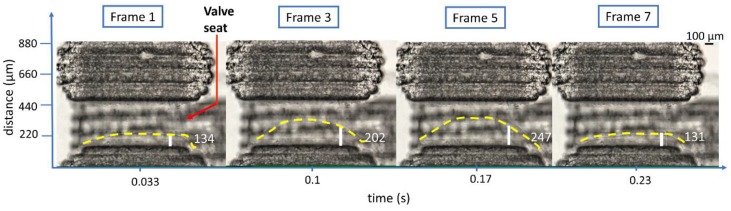
Frequency calculation using ImageJ software. The distance from an edge of the valve seat and the membrane contour (yellow dashed curve) against the PMMA surface is measured for each movie frame of the oscillation. The time interval between similar distances provides the oscillation period. Frames 1 and 7 have similar distances of 134 µm and 131 µm, respectively, corresponding to a period of six frames or a 0.2 s oscillation period (Multimedia view).

A movie of the oscillating valve was captured with a DSLR camera (Canon EOS 5D Mark II at 30 fps, Canon, Tokyo, Japan) mounted on a microscope (Nikon SMZ1500, Nikon, Tokyo, Japan); the movie was split into frames (VLC 2.1.5 media player, VideoLAN, Paris, France) for analysis. Figure 2 (Multimedia view) illustrates the frequency calculation using ImageJ (National Institutes of Health, Bethesda, MD, USA) to analyze the frames. At a fixed location from the vertical edge of the valve seat, the distance between the membrane contour (yellow dashed line) and a horizontal edge of the valve seat (referred to as contour offset) was measured for each frame. The time period of oscillations was the time interval between frames having minimal difference in contour offsets. The consistency of the value over a few cycles has confirmed the technique’s reliability.

## 3. Results and Discussion

### 3.1. Frequency Measurement

**Figure 3:**
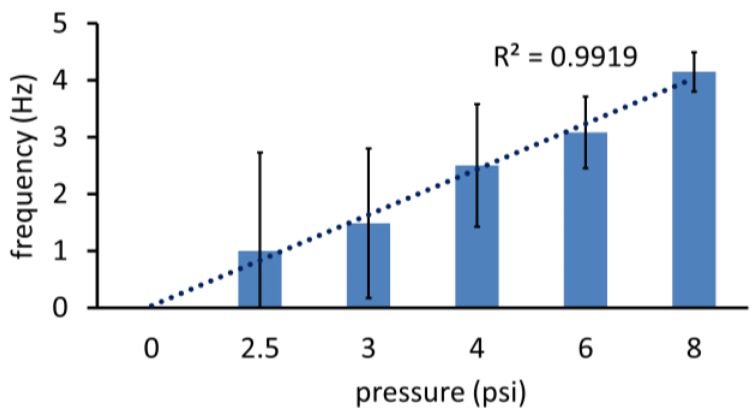
Oscillator performance with different magnitudes of constant vacuum supply. Error bars denote SD across three devices. The linear trend correlates closely with the frequency–vacuum relationship.

The variation in frequency of oscillations with the magnitude of vacuum applied (via Elveflow pressure controller, Elveflow, Paris, France) across devices fabricated and assembled in different batches is plotted in Figure 3. Frequency increases were directly proportional to vacuum over a vacuum supply range of 3–6 psi (3.08 Hz at 6 psi being twice the 1.48 Hz oscillations at 3 psi). The curve started to level off at VAC = 8 psi attaining an average 4.15 Hz, suggestive of the saturation observed in an equivalent MOSFET’s voltage transfer characteristics. A low pressure threshold of 2.5 psi was observed below which oscillations cease, corresponding to the threshold voltage required to turn on a NMOS. The frequency–vacuum supply relationship observed correlates to the alpha power law commonly used to estimate delay in CMOS circuits with short channel transistors [22], implying an analogous “short-seat” effect in the valve.

Near the vacuum threshold of oscillation (2.5 psi), a higher variation of frequency across devices was observed, indicating a sensitivity to process variations, particularly in the adhesion of the PDMS to the PMMA over the valve seat area after bonding. An examination of valve operation through the captured videos at frequencies below 2 Hz confirmed this hypothesis. As frequency increased, the lower variation across devices could be due to the adhesion being less of a dominating factor. As the sensitivity did not appear to be a limitation for higher frequency generation, the devices could be suitable for medical applications that require rapid analysis. A frequency of 4.15 Hz (at VAC = 8 psi), for example, would be useful for a controlled pump sequence.

**Figure 4:**
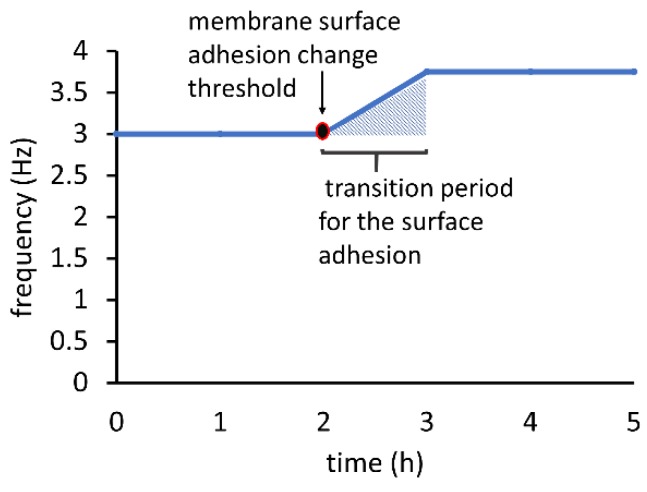
Stability of frequency with time for an oscillator operated with the vacuum supply set to 8 psi.

After observing the oscillation frequency stability over a period of a few hours, the authors made a preliminary assessment about the usefulness of the oscillator as a practical timing reference. As Figure 4 illustrates, the frequency increased by 25% after more than two hours of operation at a constant vacuum supply and stayed at the new value for another two hours. This drift implied a lowering of the threshold pressure required to open the valve, attributable most probably to the decrease in adhesion of PDMS to the PMMA over the long duration [7]. The continuous frequency increase after a period of constant oscillations suggested an apparent threshold of surface adhesion deterioration for the membrane. The deterioration continued linearly and stabilized after an hour, beyond which oscillations became constant again. Beyond the five hours of oscillations plotted in Figure 4, the frequency remained steady after further observation suggesting the possibility of long-term stable operation. After the cessation and subsequent initiation of the oscillations, the starting frequency was unaltered (pre-drift value of 3 Hz as shown in Figure 4), implying that the original PDMS adhesion was restored. This behavior reflected an initial settling time for the oscillator and supported the concept of a pre-operational running period to ensure stable oscillations for on-chip applications.

**Figure 5:**
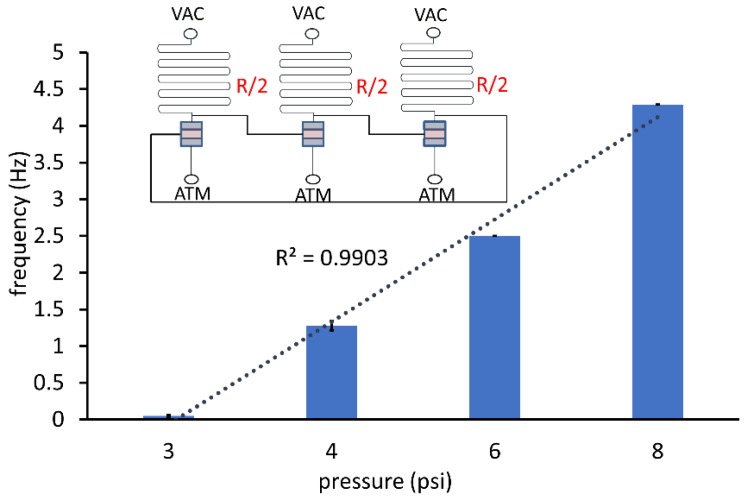
Oscillator frequency response with inverter stages having half the pull-up resistance *R*/2 (top) of the device shown in Figure 1C.

The frequency vs. vacuum pressure applied relationship for an oscillator with a smaller pull-up resistance is depicted in Figure 5. Halving the resistance did not produce a proportionate decrease in oscillation period. At VAC = 4 psi and 6 psi, the corresponding average frequencies were close to that of the oscillator with pull-up resistance *R* (see Figure 1C). A reason for the frequency remaining largely unaffected could be the contribution of the parasitic resistance of the interconnect lines (connecting the valve stages) and valve resistance dominating the overall resistance in comparison to the pull-up resistance. Reducing the valve and interconnect line lengths enabled the tuning of the frequency with the pull-up resistor length and simultaneously achieved area optimization.

### 3.2. Portability Experiment

**Figure 6:**
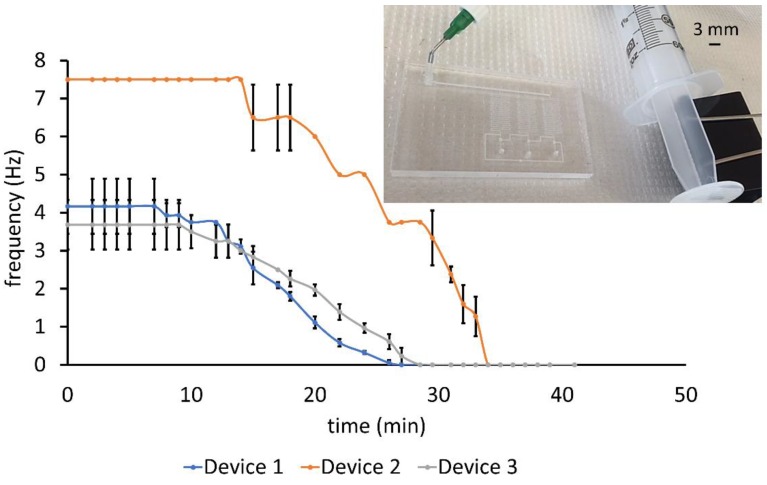
Variation of oscillation frequency with time using a syringe clamped at 47.4 mL level as the supply. Ring oscillator powered by a syringe clamped with a paper clip (inset picture). Error bars denote SD across three trials.

The portability of the device was demonstrated by powering the oscillator with a syringe. The vacuum level applied was measured by connecting a tube at the mouth of the syringe to an analog pressure gauge. Applying a steady vacuum by clamping a pulled 60 mL syringe generated oscillations that could be sustained for as long as 14 min at an average frequency of 7.5 Hz, as shown in Figure 6 which depicts the three characteristic performances of seven oscillators tested. Although the average frequency of five oscillators was between 3 and 4.2 Hz, two pulsated at approximately double the value (7.5 Hz), manifesting a property of the laser-engraving process: the effect of channel roughness on their average depth. Features made using the same raster mode laser settings on different substrates had a variability in roughness depending on the scanning pattern of the beam for the particular run. The resultant variation in average channel depth factors into fluidic resistance by an inverse cube. A depth of ~85 µm would reduce the channel resistance by almost a third and the oscillation time period by approximately 60%. 

After the initial period of steady oscillations, the eventual frequency decline observed in Figure 6 could be caused by the syringe losing vacuum. During valve closing, air was pulled into the valve chamber from the atmosphere. When the valve opened, air in the chamber flowed out through the pull-up resistor into the supply line which was attached to the syringe. The time interval for frequency drop-off to zero from the initial value was 13–14 min across devices, indicating a uniform rate of air flow into the syringe. This unvarying air flow was due to the dependency of the flow on the pull-up resistor rather than the frequency of valve operation.

### 3.3. Flow Control in Fluidic Channel

**Figure 7:**
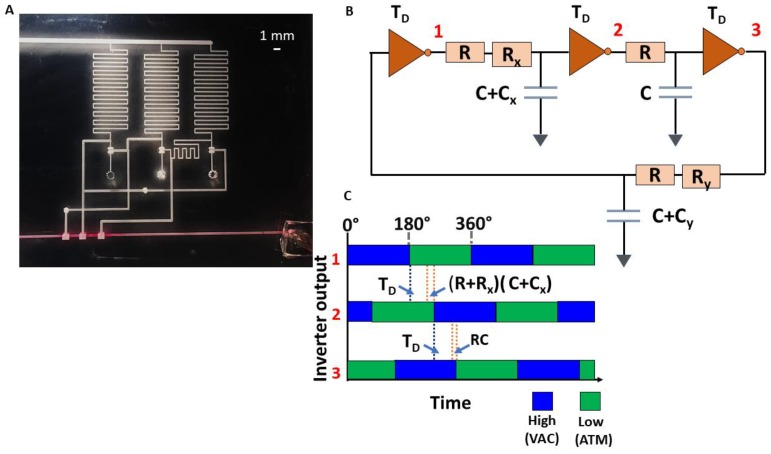
(**A**) Oscillator driving three valves in series out-of-phase in a fluid line. (**B**) Equivalent circuit for ring oscillator with varying interconnect channel lengths. T_D_ is the time delay of each inverter stage; *R*, *C* represent the resistance and capacitance, respectively, of the minimum connection line between stages; *R_x_*, *C_x_* and *R_y_*, *C_y_* represent the resistance and capacitance of the delay line of a specific length ‘*x*’ and ‘*y*’, respectively, added to the minimum connection line to obtain the necessary phase difference. In the corresponding fluidic circuit, the interconnects have negligible capacitances and the resistance primarily contributes to the phase shifts. (**C**) The phase diagram shows the shifts introduced by *R*, *R_x_* and how they affect the delay of the subsequent stage output.

A circuit wherein the individual ring oscillator inverter outputs are driving three valves in series in a fluidic channel is shown in Figure 7. The figure also depicts the relative phases of the three inverter outputs and their dependence on the time delay of each inverter stage and the interconnecting channel delay: If τ_l_ = *RC*, τ*_x_*_l_ = *R_x_C*, and τ*_y_*_l_ = *R_y_C* (*R_x_C_x_*, *R_y_C_y_*, *RC_x_*, and *RC_y_* are negligible), the equivalent time delay at the input of each stage is *T_D_* + τ_l_, *T_D_* + τ_l_ + τ_xl_, and *T_D_* + τ_l_ + τ*_y_*_l_, where *T_D_* is the time delay of each inverter. The corresponding relative phase shift at each stage is as follows: 0°, $\frac{\tau_{xl}}{T_{D}+\tau_{l}+\tau_{xl}}\times 360{}^{\circ}$, $\frac{\tau_{yl}}{T_{D}+\tau_{l}+\tau_{yl}}\times 360{}^{\circ}$.

By modifying the channel dimensions and consequently the resistance between the output of one stage and the input of another (specifically *R_x_* and *R_y_*), the phase difference can be suitably varied. The circuit serves to demonstrate the possibility of a timed sequence of opening and closing of valves, useful in an assay, for example, where specific reagents need to be mixed in at different times.

**Figure 8:**
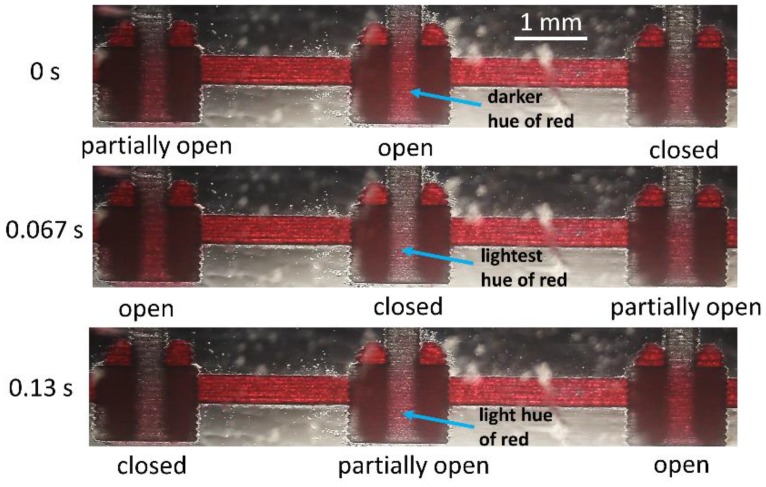
Oscillator driving three valves in series out-of-phase in a fluid line. The three phases are open, closed, and partially open. When partially open, a small volume of red-dyed water enters the flow chamber. In the open phase, the chamber is maximally filled with the water, imparting it a darker red hue. Valve closing evacuates the chamber indicated by the minimally red color (Multimedia view).

Red-colored water assisted in the visualization of the phase shifts of the pulsating fluidic valves (see Figure 8, Multimedia view). The three phases corresponding to the state of the valve in sequential order are the following: partially open, open, and closed. The time period of fluidic valve operation with a vacuum supply of 8 psi for the oscillator circuit was 0.2 s with each phase corresponding to ~0.067 s. As seen in Figure 8, the open-to-close transition was sudden, whereas the close-to-open transition was phased-out with an intermediate, partially-open state. This behavior is attributed to the elasticity of the PDMS and the strength of its adhesion to the PMMA valve seat surface.

The closed valve remained in that state due to the PDMS valve seat stiction until a threshold vacuum to open was reached. Thicker PDMS membranes were less elastic and hence separated from the rest (closed) position at a slower speed, but on the other hand, they snapped back faster to the closed state than thinner ones. With one full cycle of the valve operation equivalent to 360°, the phase difference between partially open and open/closed states was 90°. Actuation patterns depended on the phase difference between the stages and could be set by changing the channel length between stages. Adding inverter stages to the oscillator and using the output from the appropriate nodes to drive the fluidic valves is another method to achieve the desired actuation outcome.

### 3.4. Benchmarking of Oscillators

Comparing the oscillators used in the study to previously demonstrated pneumatic and hydraulic circuit implementations provided a benchmark for assessment. The semi-autonomous fluid handling by means of on-chip digital logic was initially demonstrated by Hui et al. [23]. Pneumatic oscillators fabricated with glass/PDMS and controlling the liquid pumps formed a section of the control circuitry having five external inputs. Kim et al. [24] developed micro-hydraulic oscillators using a gravity water-head as the power source that had the advantage of low-pressure actuation and enabled parallelization without pressure drops. However, a device relying on gravity is dependent on orientation and movement, affecting its suitability for portable applications. Additionally, the lower pulse frequencies achieved by such circuits (<3 Hz) versus the higher frequencies (up to 50 Hz) attainable via pneumatic oscillators are characteristic of high-resistance liquid-medium devices (resistance being approximately two orders of magnitude greater in the case of water compared to air).

Rhee and Burns [9] demonstrated digital circuits constructed with three-layer PDMS valves. Their pneumatic clock generator was implemented as a single inverter fed back through a resistance (narrow, long channel) and capacitance (chambers in the flow and control layers separated by a membrane) in series. Clock frequencies between 2 and 4 Hz for varying *RC* values and high stability over a ~2 min time period of observation were reported. One reason for the relatively short period may have been the use of a one-inverter configuration which is inherently less stable than the three-inverter structure. Loss of power in the device due to air-permeability of the PDMS flow and control layers likely contributed to the rapid decline in oscillations as well.

Pneumatic oscillators fabricated by sandwiching a PDMS membrane between etched glass flow and control chambers have been shown to work at frequencies up to approximately 5 Hz depending on the pull-up resistance, demonstrating a logarithmic drop in frequency with vacuum supply [7]. Robustness against vacuum fluctuations at small strengths has been claimed based on this behavior. The oscillators have a frequency drift of <1%/h. The authors achieved the same frequency range with a comparatively simplified fabrication process. Their oscillators showed a linear drop in the frequency of oscillations with vacuum supply with the rate of decrease becoming smaller below 3 psi vacuum, which correlated to robustness in the case of the glass-etched oscillators. Their oscillators displayed zero frequency drift for operation periods less than 2 h, which made them deployable (as an example) for analysis involving certain yeast cell cycles or measurements in cell density changes.

## 4. Conclusions

The authors fabricated a plastic pneumatic oscillator circuit that showed both long-term stability of frequency and robustness of operation, although the resolution of the laser engraver used did not allow for high-density fabrication. The out-of-phase three-valve fluidic device established oscillator suitability for self-driven control logic for microfluidic devices. A syringe was sufficient to drive the oscillator for a considerable period of time, demonstrating its applicability in portable lab-on-a-chip diagnostics. The fabrication process, based on laser engraving and APTES treatment, was reliable for the fast, efficient prototyping of these circuits during the design and development phase, as it took approximately 10–20 min to complete the laser engraving of PMMA and then four hours for the assembly. Higher-resolution laser engravers should allow for fabricating higher-density, smaller valve devices responding at higher frequencies. No additional tool adjustments or calibration is required as in techniques such as CNC milling or hot embossing. Provided that the resolution can be improved, the uniformity of features and the low cost of the process make these circuits potential candidates for simplifying the control circuitry of lab-on-a-chip devices and for driving all-plastic autonomous robots [25].
